# Transactions of the Provincial Medical and Surgical Association. Vol. XV

**Published:** 1847-07-01

**Authors:** 


Transactions of the Provincial Medical and Surgical Association. Instituted 1832. Vol. XV. New Series, Vol. III. 8vo. pp. 440, Lithographs. Churchill, 1847.

The present volume of the "Transactions" contains some interesting papers, the substance of which we proceed to lay before our readers.

### I. An Experimental Inquiry into the Effects of Hydrocyanic Acid, produced upon Animal Life. By *Thomas Nunneley,* Esq., F.R.C.S.E.

There is no circumstance more extraordinary or more distressing than the present apathy which prevails in the public mind respecting accidental or wilful Poisoning, and, although medical writers seem doomed to waste their breath in vainly endeavouring to call attention to this strange anomaly, we cannot reconcile to our conscience the omission of any opportunity of so doing. That homicidal as well as suicidal poisoning are upon the increase in this country, and that greatly so beyond what our augmented powers of detection will explain, no one who is at all conversant with what is going on in the criminal courts can be unaware: and that many of such crimes might be prevented if difficulties were thrown in the way of obtaining the instrument of destruction may at least be assumed until these have been contrived and found wanting. At present, an indirect encouragement to the perpetration of crime is distinctly held out in more ways than one. The murderer having determined upon the commission of the deed, and revolved in his mind the different modes of accomplishing it, finds that poison is pre-eminently his implement. In the first place, it is procurable, however deadly be its nature, from any chemist or country huckster, without any inconvenient questions being asked, or, if asked, readily evaded. Next, it may be so easily administered to the victim without exciting his suspicion or in directing this to an innocent quarter. Again, the symptoms its ingestion produces are so similar to, and so liable to be mistaken for, those induced by disease. It is true that the progress of modern chemical science has opposed a vast check to the hopes of escape derivable from this consideration; but while this is still so frequently at fault in the detection of poison in those cases which are fairly brought under its cognizance; and while, under the present defective means of carying on such investigations before Coroner's tribunals, so many others never come under its supervision at all, we cannot consent to deny there is still some validity in the hopes thus engendered. But lastly, the criminal may have been proved to have administered the poison which has undoubtedly caused the death, and yet the experience of several recent trials assures us that he may count upon the maudlin sympathy of his jury, who, because some one or more of their number find their opinions upon the propriety of the punishment of death clashing with the moral and civic obligations of the oath they have taken, by a curious psychical operation, sacrifice the peremptory requirements of the one to the speculative fancies of the other, and turn a villain loose upon society to perpetrate new iniquities, and to add another example of the impunity to the most deadly crimes afforded by the uncertain and capricious condition of our criminal law.

We are, however, unconsciously wandering from our proper province, our concern being here with the first only of these inducements. And in respect to this, is it too much to suppose that if the murderer, often-times impelled by passion, or the half-wavering suicide, were compelled, by the difficulty he experienced in obtaining the venenous substance, to pause in his designs, his doing so would not frequently end in his abandoning them? That it would sometimes, there can be no doubt, and this is sufficient to call for the creation of the impediments in question. And the most provoking circumstance about this matter is, that such could be contrived, without any detriment to society whatever in any other respect, and yet we go on year after year, no one seeming to think it worth his while to take the initiative. It seems to us that the following provisions might be easily carried out, and would prove effectual to the end proposed. 1. That no person should be allowed to deal in drugs or dispense medicines who had not given proof on examination of his acquaintance with the properties of the substances he intermeddles with. In the rural districts, a few common drugs, such as senna, salts, &c. might be sold, as so many of a dangerous description are at present, by chandlers and the like. 2. That even to the qualified druggist should not be permitted the unrestricted sale of poisonous substances. Some of these (as prussic acid for example) should, in fact, never be sold, except to medical men in the ordinary way of business, and if required by the public, should only be obtainable as part and parcel of a prescription. The suspicion of evil design or the fear of accident justify such restriction upon the use of poisons which are potent in their nature and only medicinal in their proper application. Others, again, of a very destructive character, (as arsenic, corrosive sublimate, oxalic acid, &c.), some of which are the more dangerous as possessing but little taste, are sometimes required for useful purposes, as the killing vermin, cleansing substances, &c. &c. But the intention of so employing them should be rendered at least probable by their purchase being made by persons competent to give an account of it, and *in the presence of a witness*. This last precaution is indispensible, for that derived from taking down name and address, &c. is a mere farce. Even when the demand of the poison is thus legitimatized, accidents should be guarded against by mixing it with other substances, where practicable, such as fat in the case of arsenic for the destruction of animals, and by selling it only in very minute quantities, just sufficient to accomplish the end in view. As to persons who apply for poisonous drugs (as laudanum, &c.) under the plea of their habitually taking them, they should be expected to make this out satisfactorily by medical or other testimony. 3. All substances of a poisonous character should not only be carefully labelled as such, but dispensed in papers, boxes and bottles of a distinctive colour. That these and perhaps additional precautions would save many, very many lives, and would, after a while, be attended with little inconvenience, we feel certain; and at all events we hope the present state of affairs, by which every needy little shopkeeper is allowed thus to trifle with human life, will ere long be remedied.

The frequency of poisoning by *Hydrocyanic Acid* is a startling fact, seeing the little excuse there is for allowing any of the public to have this deadly substance in their possession. That a chemist or medical man should now and then commit suicide by its agency we can understand; but nothing but the most culpable negligence could ever allow it to become the instrument of murder or suicide in the hands of others. That it has often become so, every one knows; and it is on this account, and because great discrepancy of opinion prevails among professional men respecting the effects which it exerts upon animal life, that Mr. Nunneley has been induced to institute a series of experiments in elucidation of these, in continuation of others which he formerly performed. They are very numerous, amounting in all to between one and two hundred, performed on dogs, cats, rabbits, frogs, fish, insects, &c.: and, from their number, the careful mode in which they were prosecuted, and the details accompanying their narration, constitute the most valuable series of facts of the description we are acquainted with. It is well known that the deductions drawn from a few experiments upon animals made at an early stage of the history of poisoning by this substance were prematurely generalized, subsequent observation having negatived some of them and shown that others were not applicable to man. It became of importance therefore, if information was to be obtained from this source, that the field in which it was sought should be sufficiently extensive. And certainly comparative experiments upon animals are justified here if they are at all, inasmuch as the effects produced upon them by the substance are so similar to those observed in man, that many valuable indications concerning the symptomatology or the treatment of this description of poisoning may be derived.

Mr. Nunneley's experiments have shown that *cold-blooded animals* do not enjoy the immunity from the effects of this acid supposed by some: although, to produce these to the same extent as in other animals, a larger quantity, and especially a longer time, are required—several minutes often elapsing before any symptom manifests itself. In one case a frog, which afterwards died, leapt the length of a foot half-an-hour after the acid had been given. Even among warm-blooded animals, in which no corresponding difference in the condition of the circulation prevails, some are more easily affected than others. "Thus the rabbit and the mouse (perhaps all rodents) are more susceptible to its action than is the cat, and the young animal of the same species than the old."

From Mr. Nunneley's observations upon the *condition of the Blood* in animals who have been poisoned by the acid, we extract the following:—

"The appearance of the blood to the naked eye is often materially changed, not only in colour, but it looks muddy and broken down, while its important property of coagulation is so interfered with that it seems to be the generally received opinion that, after death from hydrocyanic acid, the blood is almost invariably found in a fluid state: yet this is by no means the case; and unless other observers shall be more successful than myself, the examination of the blood will throw very little light upon the subject. If, however, any inference is to be drawn, it will tend to confirm the opinion that the action of the acid is upon the central nervous mass, the blood being only secondarily involved. Though the blood to the naked eye is often materially changed in appearance, yet when examined under the microscope no decided difference is seen between the globules of blood drawn from an animal before the administration of the acid and that taken immediately after death has ensued from it, whether the acid has been given by the stomach or through the lungs by inhalation. * * * * * * * It might be supposed that the action of the acid was limited to the fibrine, yet it is extremely doubtful if there be any decided change in even the fibrine, for the foregoing observations by no means confirm the idea of the fluidity of the blood after death from the acid; on the contrary, in by far the great majority of the cases the blood was found to coagulate, though by no means invariably so; possibly coagulation is somewhat delayed, and certainly in many cases the coagulum is found less dense than in blood abstracted from the body. But it must be borne in mind that, after death from almost any cause, the coagulation of the blood in the vessels is hardly ever complete, and often very imperfect as compared with that, which possibly has been abstracted from the same body only a few hours before death. * * * * So, also, though the colour of the blood is very frequently darker than natural, it is not always, as several of the experiments show. Thus, in No. 112, where out of four toads which were destroyed in a similar manner, in two the blood was florid, while in one only was it very dark; and in the cats, Nos. 89 and 98, the blood was of the brightest, most florid hue I ever saw any arterial blood, and the blood which flowed from the jugular vein of the dogs Nos. 80 and 81, was certainly not darker than usual." P. 68.

The author delivers a useful caution to experimenters in these days of hasty microscopic deduction, viz. to examine the healthy blood of the same or of corresponding animals. Having found the globules of the blood of a kitten who had been poisoned to be for the most part of a hexahedral or octahedral form, he had nearly attributed this to the effect of the acid, when, upon examining the healthy blood of other kittens, exactly the same shaped globules were observed, these being at once converted into circular ones by the addition of a minute portion of water or of the acid.

In an Appendix, Mr. Nunneley gives an account of some experiments he subsequently made by injecting the acid into the external jugular veins. Of these he observes:

"These experiments sufficiently prove, I think, that hydrocyanic acid does not produce any other effect when injected into the blood than when administered in any other way, the only difference being a somewhat speedier effect, and also, I think, somewhat more decided, so that probably a rather smaller quantity would destroy life by injection than when applied upon a membrane; though, when all circumstances are taken into consideration, I doubt whether absolutely the same quantity coming into *actual* contact, over an equally large surface, upon a mucous membrane, whether alimentary or respiratory, or even the conjunctiva, would not produce, if not as speedy, at least as decisive effects as when injected into the blood. They also confirm what has been stated as to the acid not acting upon the structure of the blood, for here not any difference could be seen in the characters of the blood; its colour and coagulation were as usual." P. 370.

In a medico-legal point of view it is of importance to remember that

Mr. Nunneley's experiments show that the acid acts with nearly if not quite as much rapidity and certainty when applied to any of the mucous membranes, as the vagina, rectum, or conjunctiva, as when swallowed. Other experiments prove, as indeed accidents had already done, that the inhalation of air impregnated with it is another certain and rapid mode of poisoning by it; "one which it would be very easy to employ, but most difficult after a few hours to detect, as the odour, being so diffusible, is very soon dissipated." Its application within the meatus auditorius and to the external surface of the body has produced but little effect.

The following are Mr. Nunneley's opinions upon the mode in which this poison influences the *action* of the *heart*.

"I do not agree in the opinion which has been expressed by some (Dr. Lonsdale, Ed. Journal, Vol. 51), that the heart only ceases to beat because of the suspension of respiration, and its consequently only containing dark blood. It rather appears to me that the cause of death is altogether different from what occurs in asphyxia from the occlusion of the air-passages, the heart being primarily, and to at least an equal extent, affected as respiration. This is not merely a theoretical question, but a most important practical one, as it must materially influence the treatment. If the heart's action only ceases on account of the want of respiration, and the consequent engorgement of its cavities with dark blood, venæsection would be one of the remedies to which we should first have recourse, and from which we should expect the most decided benefit; while, on the contrary, if the state of things be as I suppose—not only that the blood itself is changed by the introduction of the poison, but that the heart is primarily affected, our reasons for having recourse to it would be much less powerful; neither should we expect the same amount of benefit from its employment." P. 72.

After stating that, although the experiments showed little benefit was derivable from venesection, that where congestion occurs it may be usefully, if moderately employed, he continues—

"Venæsection may, therefore, in some few cases, in which the dose of the acid has been small, be proper and advisable enough, when practised with the understanding of why we employ it, and too much blood be not abstracted;* but as there is great loss of power as well as spasm at the time, and where recovery takes place considerable weakness and depression are for a while manifested, the taking away a large quantity of blood, in my opinion, is likely to be rather injurious than beneficial. The condition of the heart, at least at first, is one of contraction, not of dilatation. There is a persistence of contraction, not a diminution of contractibility, so long as the spasm continues, and of the left cavities, for long after. The cavities, it is true, may possibly occasionally afterwards become engorged, but this is only when the heart, like the general muscular system, becomes paralysed, and certainly is not supported by the condition of things revealed by the post-mortem examinations above recorded." P. 73.

The author concluded at first, with other observers, that the evidence derivable from post-mortem examination in this description of poisoning is merely negative, so contradictory have the reports furnished been: but,

---

* "Of this, in most cases, there is little danger, as the blood will not flow. Indeed, I am not sure the distress in respiration is not in a great measure caused by the want of blood passing through the lungs. It appears very probable that but very little blood is passed forward by the heart."

when he examined the body immediately after death, he found the explanation of the different statements made in reference to the state of the heart and vessels. When death is long delayed, or the dose of the acid very small, the blood is usually dark, and all the cavities of the heart may contain more or less of it, especially the right, which is often much distended. If the death had been sudden, the left side of the heart, and especially its ventricle, was almost always found perfectly empty and rigidly contracted, the right side being at the same time, though not always, much distended. In nearly every instance the aorta and its large branches were, like the left ventricle, found empty. As shewing that this different condition of the two sides of the heart is not dependent upon obstruction of the lungs the detailed post-mortem examinations are referred to.

"This condition of the heart and great vessels sufficiently explains, what has given rise to much surprise, why it is that with the venous system so much congested so little blood should flow on a vein being opened. It is obvious that it cannot. The circulation is positively suspended; though there be motion of the heart, the blood is not propelled; there is no *vis â tergo*. For, as the left ventricle will not open to receive the blood from behind, so it cannot give the onward impetus; hence, should the blood in some degree flow, or afterwards continue to drain away, this will merely depend upon the circumstance of the aperture being in the most depending position, when, if the blood continues fluid, some of it will find an exit, as any other fluid does from an opening below its level, a mode of escape altogether different from that of ordinary venæsection." P. 77.

The author's subsequent observations, made while injecting the acid into the veins, also confirmed this view. In a minute, or a minute and a half, blood almost ceased to flow from even such large vessels as the external and internal jugulars.

Very erroneous ideas at one time prevailed as to the *rapidity* with which the effects of this poison are produced, and the possibility of certain voluntary acts being performed subsequent to taking it. Many authentic cases now prove that its action is by no means so instantaneous as once supposed. Mr. Nunneley believes some of his experiments tend to show that consciousness may, when the dose is not very large, be retained, after all voluntary power is extinct, and but little sensibility remains. This would indicate the spinal marrow as being more obnoxious to its influence than other portions of the nervous system, an inference likewise derivable from the violent spasmodic action alternating with paralysis which is produced. However this may be, the experiments confirm the statements now usually received of the length of time during which both consciousness and volition may be retained; for, although in a few instances the action of the poison was too rapid to admit of a manifestation of these, in most of the dogs and other warm-blooded animals about *twenty seconds* elapsed ere any symptoms were manifested, and in several a much longer period. There does not seem to be any *fixed quantity* of the acid which will invariably destroy life, much depending upon individual and varying circumstances. The more vigorous the animal, *cæteris paribus*, the larger is the quantity required. A full stomach lessens the effect, and age exerts a material influence. Mr. N.'s experiments seem to show that an animal is easily brought under the influence of the poison, just in proportion as it is young; within however certain limits, for if it be *very young*, a larger dose is required than is necessary to poison one of the same species a little older. "This is so curious a fact, that were there not sufficient evidence to support it, we should feel much inclined to doubt it. Is it to be regarded as another proof of the approximation of the young of the higher species to the adult of the lower?" The degree of *concentration* of the acid employed exerted no very material influence—a moderate degree of dilution, in some cases, seeming to have augmented its effect. Neither did death follow with rapidity in proportion to the *quantity* taken, supposing the minimum dose to suffice for speedy death. Thus, supposing 40 m. of Scheele's acid kills a dog in four minutes, it does not follow that 80 m. or more would do so in half the time. "Hence, when called to a person poisoned, we cannot, merely from the length of time he has survived, or the violence of the symptoms, determine anything with certainty as to the degree of concentration or dilution of the acid, nor, except within wide limits, much as to the absolute quantity taken."

Too much reliance was at first placed in our criminal courts upon the occurrence of the *death-shriek*, as symptomatic of this description of poisoning in man. Mr. Nunneley's experiments show that it is as often absent as present even in dogs, and in not more than one-third of the number is it very loud. When it does occur, however, it is entirely and distressingly peculiar. "It is different from any thing I have heard in any other condition of dogs or other creatures, and is, I think, when present, characteristic of the poison."

*Treatment of Poisoning.*—Mr. Nunneley availed himself of the opportunities which his numerous experiments presented him with, of examining this important part of the subject; but with very defective results, as respects the discovery of an antidote, or even the treatment of the effects of the poison. Chlorine, alkalis, and the preparations of iron, were found unavailing for the first of these objects. For counteracting the effects electricity was of no avail, if it did not aggravate these. Cold affusion, which has been so much lauded as a means of arousing the energies of the system, is thought of little avail by Mr. N. in any but the slighter cases of poisoning, and in these only when used with discrimination, and not too prolonged. He observed that shaking the animal briskly seemed to exert a marked beneficial effect in several cases. In bleeding, we have already seen, he has but little faith. Emetics, although immediately given, were of no avail whatever, when the dose of the acid had been large; but when this has been but small, or taken into a stomach already containing other ingesta, they may be useful. Owing to the favourable reports which have been published concerning the effects of ammonia, Mr. N. put these very freely to the test of experience. This convinced him that its efficacy has been exaggerated. Applied externally it was not of the least avail. "I think all that can be fairly adduced in favour of ammonia is, that as it may be beneficial we ought to have recourse to it, not with the expectation of deriving benefit from it in severe cases, but that in those cases where the dose has been so small as to render it uncertain which way the balance will turn, it may assist it to incline on the side of life."

### II. Observations on the Operation of Ovariotomy. By *George Southam*, Esq.

Mr. Southam here presents us with the details of a case in which this operation was unsuccessful, adding to these some interesting remarks. He truly observes that, it is a duty of one who has recordad successful cases to give an account of those in which he fails, a duty, however, we fear, especially as regards the operation in question, very imperfectly fulfilled. "The suppression of the particulars" by such practitioners, he observes, "can only be regarded as a proof that the operators thought they would reflect no credit on their judgment, either in a medical or surgical point of view. These ought not, therefore, to be adduced as objections, the fault being with the operator rather than the operation." This conclusion is scarcely correct. In ordinary operations we do not find the same refusal to publish unsuccessful cases; and we cannot but think that fear lest the medical public would regard the undertaking this one, in the cases in question, as utterly unjustifiable, has prevented disclosures so morally obligatory and vitally important for estimating the true position of ovariotomy as a legitimate operation, unsettled as this is at present amidst the crude statistical statements which have been adduced. Death from other operations does not, at least only in a very insignificant proportion of cases, occur in consequence of the case having been wrongly diagnosed, or the operation abandoned without being completed—circumstances avowedly of frequent occurrence in respect to ovariotomy. We see no reason to alter the opinions expressed in a former Number of this Review,* and we entirely doubt the accuracy of Mr. Southam's statement that, "by a majority of those who have made the diseases of females their more especial study, ovariotomy is now considered as perfectly justifiable." We believe exactly the contrary is the fact, and that few of our eminent obstetricians have so expressed themselves; and even if they had, we must appeal to a more competent tribunal, and one more accustomed to estimate the difficulties and calculate the chances of surgical operations in general; and we are aware of the names of scarcely any of our operating surgeons who have as yet admitted ovariotomy as a justifiable undertaking, except in very rare and exceptional cases.

We need state only a few of the particulars of the case. The patient was 26 years of age and had had five children. The tumour was first perceived ten months prior to the operation, had rapidly increased in spite of bandaging, frictions, and was complicated with ascites. Its diagnosis from uterine disease was a matter of some difficulty—Dr. Simpson's sound obscuring rather than aiding this. As a difference of opinion prevailed concerning the origin of the tumour, Mr. Southam made an incision but three inches long, mid-way between the umbilicus and pubes, until he had ascertained that it was unconnected with the uterus, when this was prolonged above the umbilicus, and the tumour, which was found connected to the broad ligament and posteriorly to the omentum, removed without much difficulty. The operation was completed within twenty-five minutes, the

* Med.-Chir. Review, N. S., Vol. I., pp. 26 and 42.

patient being repeatedly faint, although little blood was lost. The woman lived until the sixth day after the operation, sinking apparently from want of constitutional power. The post-mortem examination exhibited slight traces of recent peritonitis; and the pedicle of the tumour, which had been encircled with the vessels in the ligature, was in a state of complete sphacelus. "The risk from the operation would, no doubt," Mr. Southam observes, "be much diminished if the vessels could be alone secured, which I believe is practicable in a majority of cases, as there are seldom more than three that require tying."

The question of the *malignancy* of ovarian tumours forms an important element in considering the propriety of their removal. It can be best decided by watching and recording the condition of patients who have undergone the operation of extirpation; for we quite agree with Mr. Southam, that the microscope is at present a very unsafe guide in this matter. Of this opinion are several of our most eminent microscopic observers, among whom is Mr. Goodsir, who, reporting to the author upon a specimen of the tumour submitted to him, says:

"I put very little value on the microscope in detecting the nature of tumours of any kind. It is by what we call in natural history the *habit*, or *general appearance*, or *bearing*, of *plant*, *animal*, or *morbid growth*, that the *species* or *nature* in doubtful cases, is to be determined. In disease this tact in discrimination is to be acquired not by poring through a microscope, but by experience in the *diagnosis* and *handling* of tumours in the living, and the examination, by the naked eye, of their general appearance in the dead. The microscope can only *verify the previous determination.*" P. 103.

We cannot admit that this grave operation was justifiably performed upon a woman of enfeebled frame, the subject of ascites; but Mr. Southam's narration of its unsuccessful issue is entitled to much praise.

### III. Observations on the Pathology of Abscess of the Heart. By *T. H. Stallard*, Esq.

Mr. Stallard details an interesting case of this. A shoemaker, æt. 60, and previously very well, was seized, 22nd March, while at work, with coma, cyanosis, and great prostration—his pulse (60) being full, soft, and feeble, and the respiration slow and gentle. Stimuli were given him, and next day he rallied somewhat, the pulse becoming stronger, and the respiration a little quicker. On the 25th he was thought to be asleep, but was found to be dead.

"On opening the left ventricle, which was accomplished by a V-shaped incision, an abscess was observed. It was situated at the apex of the ventricle, and of a very irregular shape, being most pointed towards the apex of the heart, from the surface of which it was separated by two or three lines of healthy structure; above, it projected considerably into the cavity of the ventricle, with which it communicated by a small fissure; the interposed septum was about one line thick, and appeared to consist of thickened endocardium. The cavity of the abscess contained a bloody, purulent-looking fluid; its lining membrane was of a light red tint, and presented a granular appearance. Around the abscess the muscular tissue was darker than natural, and in the outer wall of the same ventricle were observed several fissures, containing a dark-coloured fibrinous material, and some of these, though not all, communicated with the cavity of the ventricle. The coronary arteries were much ossified." P. 107.

Mr. Stallard argues the improbability of this abscess having arisen from acute or chronic carditis, since all symptoms of this were absent, and no signs of the always accompanying endocarditis or pericarditis were found after death. The dark fibrinous matter found in the substance of the ventricle had all the appearance of coagulated blood. The presence of blood in this situation may be explained, 1, by the fluid being forced between the *columnæ carneæ*, so as slightly to rupture the endocardium. 2. Congestion of the coronary vessels may terminate in rupture. 3. A partial rupture of the heart's muscular fibres may occur—and this the author regards *as a frequent cause of angina pectoris*. Blood effused from any of these causes may be slowly re-absorbed, remain for a period unchanged; or, if in sufficient quantity to interfere with the action of the organ, the constitutional powers being at the same time feeble, it may undergo the changes so ably pointed out by Mr. Gulliver,* and become softened into a puroid fluid, which, however, resembling pus in apppearance, is easily to be distinguished from it by the aid of chemistry and the microscope.

"I have thus endeavoured to show that the case I have related is one dependent upon the effusion of blood into the substance of the heart, that the abscess resulted from softening of the coagulum so effused, and that this having possibly existed for some considerable time, at length escaped into the ventricular cavity, and caused slow death by poisoning the circulating fluid. I shall now proceed to confirm this opinion by one or two recorded cases, and whilst my observations will tend to show that carditis, or myo-carditis, is more rare than is generally supposed, I by no means assert that it never takes place. Andral cites several cases to show that redness of the muscular substance, observed after death, had been caused by an attack of carditis, and this is also the opinion of Dr. Elliotson: but it is remarkable that, in all these cases, severe symptoms of cardiac disease preceded death, whereas none, or few, were present in cases specially related as instances of pure carditis." P. 111.

An abridged account is given of two cases of abscess supposed to arise from carditis, related by Mr. Salter in the *Medico-Chirurgical Transactions*,† (Vol. 22), and by Mr. Chance in a recent number of the *Lancet*.‡ Mr. Stallard believes the symptoms and post-mortem appearances in these are best explained by the above hypothesis; and observes that the point is an important one, as a correct plan of treatment can only be founded upon a proper comprehension of the true pathology of the disease. In the one case antiphlogistic means would be called for, even though the symptoms indicated prostration, while in adopting Mr. Stallard's views, stimuli are indicated upon the following grounds:—

"1. To stimulate the vital energies, in order to prevent the effused blood becoming softened and decomposed, and thus to favour its absorption or organization. 2. If the clot shall have already softened and entered the blood, to obviate or at least stave off the effects of poisoned blood, which alcohol has a great power of effecting. 3. Should the fluid have escaped into the pericardium, to stimulate the system to the production of pericarditis; which, however undesirable under ordinary circumstances, is in this instance curative, since it closes the opening

---

* See Med.-Chir. Transac. Vol. 22, p. 151, or Med.-Chir. Rev., No. 63, p. 47.

† See Med.-Chir. Rev., No. 63, p. 51. ‡ No. 1185.

of the cavity, and strengthens the parietes of the heart at a point peculiarly requiring support." 116.

Mr. Stallard has, in these observations, drawn attention to a very interesting subject, well worthy of the renewed attention of pathologists.*

VI. An Essay, Literary and Practical, on Inversio-Uteri. Part 2. By *John Green Crosse.*

This is a continuation of the elaborate monograph we have already given some account of,† and is marked by the same laborious research which characterized its predecessor. It is, however, more interesting and original than that, and will be perused with advantage. Mr. Crosse describes several stages or degrees of inversion, viz., simple *depression* of a portion of the fundus, which may give rise to a severe or even fatal hæmorrhage. It soon passes, however, into the state of *introversion*, in which the fundus and a portion of the body of the organ is "received into the remainder of the body and cervix, the convexity of the fundus being palpable at the os tincæ." *Perversion* is said to exist when more or less of the inverted portion passes through the os tincæ. In *total inversion* the cervix, as well as the rest of the uterus, is inverted. The former part of the Essay comprised a description of the varieties of the disease and their distinctive symptoms, and the present one commences with an account of the author's opinion of the alleged

"*Infrequency of Inversion.*"—According to statistical evidence, this disease is of very rare occurrence. Thus, of Mauriçeau's 850 cases selected for publication, only six were examples of this. Dr. Collins met with no example in 16,654 deliveries at the Dublin Lying-in Hospital, while Dr. M'Clintock, speaking of that hospital, states that no case was met with amidst *seventy-one thousand* deliveries. A lying-in hospital is not, indeed, the place we ought to expect to meet with an occurrence which generally arises from mismanagement. However this may be, the disease is by no means so rare as the above statement would show, many authors, whose works are cited by Mr. Crosse, relating several cases. Altogether he has been enabled to collect 400 cases, of which 350 occurred after parturition, 40 from polypus, and 10 from various other causes. The belief in the great rarity of the disease is calculated to favour the overlooking of its minor degrees. These have been observed by several of Mr. Crosse's medical friends since he has so particularly directed attention to the subject; but he believes that uterine hæmorrhage still frequently occurs without partial inversion being suspected as its cause. "No other case," he observes, "has, in relation to the infrequency of its occurrence, been so often brought into a court of justice as *inversio uteri*;" and in truth the instances, some of which of recent occurrence he cites, in which not merely ignorant midvives, but practitioners supposed to be qualified, have mistaken, mutilated, or removed this organ, are frightfully numerous.

---

* See Med.-Chir. Rev., No. 35, p. 174, and N. S., No. 9, p. 40.
† Med.-Chir. Rev., N. S., Vol. 2, p. 467.

The perpetrators of such outrages, who have sometimes been screened by well-meaning medical men, should be placed beyond the pale of professional sympathy, as, although our law courts seem little competent to deal with such delinquents as they deserve, the exposure of their misdeeds, and the consequent loss of reputation this entails, is a debt we owe to the public; for, although we should be careful not to render patent the errors our brother-practitioners may fall into in the exercise of a fallible judgment, we should refuse to place in this category those results of ignorance so mischievous and glaring as to render the pursuit of the profession by their authors a matter dangerous to society. There is a limit beyond which our duties as members of society are paramount to all considerations of medical etiquette.

Passing over the sections upon the symptoms and pathology of the disease, we come to that which treats of—

*The Causes and Mechanism of Inversion.*—Causes of a contradictory and insufficient character have been assigned as competent to the production of Inversion. *Predisposing Causes* may exist long prior to the occurrence of the accident, and among these may be classed whatever tends to enfeeble the system, inversion, in the majority of instances occurring in women of delicate frame and relaxed fibre, with such a loose state of the pelvic soft parts as usually yields to a quick or precipitous delivery. The erect posture is favourable to its production, many instances having occurred in which the woman was suddenly delivered before she could assume a recumbent one. *Over-distension* of the uterus, as by the *liquor amnii,* has been supposed to dispose the organ to this accident. Mr. Crosse's investigations do not corroborate this view; for, in such case, we should expect the accident to more frequently follow the birth of twins, while of the 400 cases he has collected, only four were examples of this. Moreover the accident is of far more frequent occurrence in the young primipara than in women who have already borne children. He believes that some of the worst cases have depended upon great *thinness* or defective density of the uterine parietes; but he regards a derangement of the normal vital action of the organ, rather than its different mechanical properties, as the usual predisposing cause—a *partial inertia* or *a want of contraction* inducing the *depression* or first stage of the displacement.

*Relaxation of the round ligaments* has been classed among the predisposing causes, and they are at all events passive and incapable of affording resistance. The *previous occurrence* of the *disease* seems to have taken place in very few cases. *Hæmorrhage,* generally dependent as it is upon partial inertia of the uterus, may act as a predisposing cause by increasing that inertia; but, if the blood has accumulated in the cavity, its sudden escape may be attended with inversion. The *attachment of the placenta to some part of the fundus uteri* seems to be almost an essential condition for the production of the disease, and may be regarded as forming a connecting link between the predisposing and determining causes. But neither this or any other of the predisposing causes have much effect, unless several of them be conjoined.

The *immediate or determining causes* of inversion may be classed under three heads. "1, Such as are situated in the uterine tissue itself; 2, such as act upon the outer surface of the organ, pressing or driving it down; and 3, such as act upon its internal surface, drawing or pulling it down." Though not competent to *commence the displacement causing depression*, the uninverted portion of the *uterus* will act upon the portion already incipiently inverted from any cause, as upon an extraneous mass, and complete the inversion.

The *expulsive abdominal nisus* of the patient may much aid in the production of depression, and afterwards assists in every stage until complete perversion; and it is indeed sometimes the most active power in the production of the worst cases of rapid inversion. In many recorded cases we find the inversion to have been latent or unsuspected for hours, or a day or two, until some expiratory effort has increased it to perversion and prolapse. In the third class of causes, *violent traction through the cord*, has often induced the accident; but in an enfeebled constitution, and when the predisposing causes are numerous or considerable, the slightest traction by a careful practitioner may suffice to induce the first degree of the affection. The weight and even the bulk of the placenta, by distending the lower part of the uterus and exciting its expulsive action, may thus contribute to the completion of the inversion. A *short funis*, or one that is coiled around the body of the child, may render depression unavoidable when the placenta is attached to the fundus. The *too rapid removal of the body of the fœtus* from the uterus, after the birth of the head, will, "upon the principle of a tendency to a vacuum (?), draw down the relaxed fundus." *Morbid adhesion of the placenta*, by the encouragement it affords to traction at the cord, may also cause inversion. Not only must the predisposing and efficient causes unite for the production of this displacement, but there is usually a plurality of each contributing to this.

Mr. Crosse has an excellent chapter upon the *Diagnosis* of the Disease, and treats it more minutely than any other writer we are acquainted with. We have only room for one or two of his general remarks.

"In no instance has the importance of a correct diagnosis been more strikingly illustrated than in uterine inversion, which has been mistaken more often, in proportion to its frequency, than any other malady of its class, and, perhaps, of *any class*. These mistakes have usually been attended with the most fatal effects, for many are the examples, attested by the best authorities, where it is observed that, had the nature of the case been timely known, it would have been rightly treated, and life easily saved.

"In commencing this section by considering recent inversion, I find reason to remark that errors in its diagnosis have frequently been but hasty and inconsiderate impressions, which a moment's reflection would remove; or they have arisen from the want of previous experience, and the absence of all suspicion, owing to the real or supposed rarity of the displacement. * * * * * * The short and dogmatical rules of some of our most esteemed writers, would indicate the diagnosis to be very easy; the authentic records of our art seem to show the contrary, even as regards recent inversion post partum, which in its earlier stages has been mistaken for the head of another fœtus, another placenta, a mole, an excrescence, a polypus, a tumour, and a clot of blood; and, in its more advanced stages, for not only some of these, but for still more unaccountable diseases and displacements. It is very difficult to explain the source of all these erroneous impressions, which not only midwives, but even well-educated and practised surgeons have acknowledged, until we consider that the emergency is great and sudden,—some opinion or other must be rapidly formed, and those ideas with which the mind is more familiar first present themselves. The pressing urgency of the circumstances is even greater than the surgeon estimates them to be, for uterine inversion, at the close of the delivery, is a case that leaves little time for consideration, none for preparation, ere a decision must be arrived at, or the risk be incurred of the patient's expiring of her undetected, or misunderstood, and consequently maltreated, disorder." P. 317.

*Prognosis.*—No disease of its class is more fatal than this, unless effective aid be promptly rendered. The mortality from it has been variously estimated; but Mr. Crosse, from the most extended investigation yet brought to bear upon the subject, is in a condition to affirm that "above one-third of all the cases, under whatever circumstances, or in whatever degree they occur, prove fatal very soon, or within one month after the displacement is produced." Of 109 fatal cases, collected from various sources, in 72 death took place in a few hours, generally within an hour and a half, and often nearly immediately. Eight cases proved fatal in from one to seven days, and six in from one to four weeks. "It deserves to be forcibly noted that, although 86 cases were thus brought to a close within *a month,* this period being passed, only one of the remaining 23 proved fatal before arriving at the chronic stage," the remaining 22 terminated at different dates extending to three or four years, and in a rare case or two, to 5 or even 20 years. The less the degree of inversion when we are called to the case, and the slower it has advanced, the smaller the amount of danger. The organ may perhaps be so relaxed as to admit of ready reduction, though entirely inverted; but this may arise from the patient being already *in articulo.* A rapid spontaneous inversion may be more dangerous than one inverted by traction of the cord, as the patient in the latter case may possess a strong constitution, enabling the disease to go on to its chronic stage. In the stage of depression the consequent hæmorrhage, if the displacement be not rectified, may alone kill the patient, but when the organ is extruded, we have the influence of "shock" superadded to this. Mr. Crosse believes that total inversion can only be rectified by almost immediate attempts, but the patient has a better chance of recovery by this remaining unattempted than from its accomplishment through violent means.

We regret that our limited space has prevented our furnishing a more complete account of Mr. Crosse's interesting Essay, and hope that he will not long delay the publication of the concluding portion, embracing the treatment of this formidable accident and its consequences. The same reason likewise precludes our noticing a paper by Mr. Barker, describing the Removal of a *Large Secondary Prostatic Calculus* by Perineal Incision; the *Retrospective Address* of Dr. Ranking; and a short Essay by Dr. Paxton upon "*Pathological Memorials,*" in which he ably advocates the desirableness of making pathological drawing part of medical studies.

Upon the whole the volume is an excellent one, and well illustrated by numerous lithographs.

